# Is Chest Wall Resection Safe in Geriatric Non‐Small Cell Lung Cancer?

**DOI:** 10.1111/crj.70137

**Published:** 2025-11-10

**Authors:** Ozkan Saydam, Celal Bugra Sezen, Melike Ülker, Umut Kilimci, Oguzhan Bayraktar, Mustafa Vedat Doğru, Cemal Aker, Muzaffer Metin

**Affiliations:** ^1^ Department of Thoracic Surgery Yedikule Chest Diseases and Thoracic Surgery Training and Research Hospital Istanbul Turkey

**Keywords:** chest wall, non‐small cell lung cancer, prognostic factors, resection

## Abstract

**Purpose:**

Our aim in this study is to evaluate the safety and efficacy of surgery in patients undergoing chest wall resection due to non‐small cell lung cancer (NSCLC) based on age groups.

**Methods:**

The study was conducted retrospectively on 160 patients with NSCLC who underwent chest wall resection between 2009 and 2019. Patients were classified into Group A (under 70 years) and Group B (70 years and older).

**Results:**

The study found a complication rate of 28.1%, but no negative impact of the geriatric age group on complications was determined. The risk of complications varied depending on the number of ribs removed (*p* = 0.035). The survival rate for Group A was 72%, while for Group B it was 93% (*p* = 0.189). No significant differences were found in terms of gender, Charlson Comorbidity Index (CCI), and histopathological results. In patients who underwent lobectomy, survival was 85%, while a significant difference was observed in those who underwent pneumonectomy, with a survival rate of 41% (*p* < 0.001).

**Conclusion:**

It was determined that advanced age is not a prognostic factor in surgical resection regarding complications and survival, with the most important prognostic factors being the type of resection and the stage of the disease.

## Graphical abstract

Chest wall resection can be performed safely, even in cases involving extensive invasion, including wide chest wall tumors. Our findings support the feasibility and safety of en bloc resection and reconstruction in appropriately selected elderly patients. Perioperative images demonstrating the reconstruction of the extensively invaded chest wall are presented in the figure.

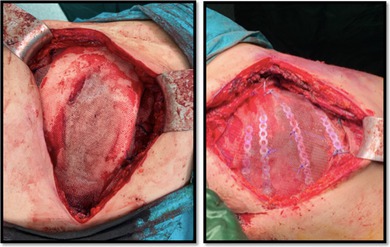

## 1 Introduction

Patients in the geriatric age group with non‐small cell lung cancer (NSCLC) face numerous challenges during their oncological treatment processes. Lung cancer is one of the most commonly diagnosed cancer types globally, particularly prevalent among geriatric individuals. In treating early‐stage NSCLC, surgical intervention is currently considered the gold standard treatment protocol [1, 2]. In cases of chest wall invasion due to local tumor invasion, thoracic wall resection plays a significant role in lung cancer treatment. However, elderly patients often encounter various comorbidities and challenges related to their overall health status alongside cancer. This situation increases the risks associated with lung cancer treatment in geriatric patients and raises questions about the effectiveness of surgical interventions. Due to the risks associated with advanced age, surgery for locally advanced tumors remains a controversial topic [3].

Chest wall resections in geriatric patients require special attention concerning postoperative complications, recovery processes, and overall quality of life. Physiological changes associated with aging may affect the response to surgical interventions. Additionally, the presence of existing health issues and low functional capacity in most elderly individuals can increase the risks of surgery. Studies conducted in the literature involving the geriatric population have indicated that patients achieve acceptable oncological outcomes; however, it has been demonstrated that the width of resection does not affect early and late oncological results, while larger resections increase the risk of postoperative complications and mortality. In this context, it is argued that limited resections should be performed to reduce the risk of postoperative complications [4, 5]. It has been shown that limited resections (e.g., segmentectomy) yield survival outcomes similar to lobectomy, emphasizing the effects of the size of surgical resection and radical lymph node dissection on survival.

Recent studies in geriatric oncology highlight the importance of multidisciplinary approaches in the treatment of elderly patients [6]. In addition to surgery, personalized approaches to oncological treatment can help improve the general health status of geriatric patients and enhance their responses to treatment. Therefore, the potential benefits and risks of chest wall resections in the geriatric age group should be carefully evaluated. This article will assess the safety and efficacy of surgery in patients undergoing chest wall resection due to NSCLC according to age groups.

## 2 Methods

This study was conducted on patients with locally advanced NSCLC who underwent surgery at our clinic between January 2009 and December 2019. The study included patients who underwent en bloc chest wall and lung resections. The study was designed retrospectively. Patients' surgeries performed contrary to oncological principles (wedge resection patients) and those who did not undergo systemic lymph node dissection were excluded from the study. Ethical approval for the study was obtained from the local ethics committee of our hospital.

### 2.1 Patient Selection

In the preoperative period, all patients underwent routine thoracic computed tomography (CT) to evaluate the location and invasion of the tumor. Positron‐emission tomography (PET) and magnetic resonance imaging (MRI) were used to detect metastases. All patients underwent endobronchial evaluation via preoperative bronchoscopy performed by surgeons. Mediastinal evaluations were performed using endobronchial ultrasound (EBUS) and/or mediastinoscopy. The modified Charlson Comorbidity Index (CCI) was used to assess the impact of comorbid conditions on mortality and complications. Patients were divided into two groups: those aged 70 and younger (Group A) and those aged 70 and older (Group B). Postoperative complications were considered events occurring during the hospital stay or within the first 30 days after surgery. Mortality was recorded as deaths occurring within the first 30 days after surgery. Demographic data, morbidity, length of hospital stay, mortality, and histopathological characteristics of the patients were analyzed. Pathological staging was conducted according to the 8th edition of the TNM classification system. Follow‐ups for patients in the postoperative period were conducted with physical examinations and radiological imaging every 3 months for the first 2 years, every 6 months between the second and fifth years, and annually after the fifth year.

### 2.2 Statistical Analysis

Statistical analyses were performed using SPSS Version 22.0 (SPSS Inc., Chicago, IL, USA). The chi‐square (*χ* ^2^) test was applied to calculate frequency values for descriptive statistics. The *T*‐test was used to compare means between independent groups, while the Mann–Whitney *U* test was used to compare medians. Survival analysis was performed using the Kaplan–Meier method, and curves were compared using the log‐rank test. Multivariate analysis was performed using Cox regression. A statistical significance level of *p* < 0.05 was set.

## 3 Results

A total of 160 patients were included in the study, of which 152 (95%) were male and eight (5%) were female. The average age of the patients was 66 years (age range: 41–90). One hundred fourteen patients (71.3%) were under 70 years old, while 46 patients (28.7%) were 70 years old or older. Eighty‐five percent of the patients (136 patients) had a history of smoking, with an average smoking duration of 30 pack‐years (range: 0–62 pack‐years). There were 124 patients (77.5%) with a CCI of “greater than 4,” while 36 patients (22.5%) had a CCI of “4 or less.” Surgical procedures performed included left resection (40.6%) and right resection (59.4%), with 136 patients (85%) undergoing lobectomy and 24 patients (15%) undergoing pneumonectomy. Mesh (polypropylene mesh) was used in 20 patients (12.5%). Additionally, 59 patients (36.9%) received neoadjuvant therapy.

When examining the T stages of the patients, 13 patients (8.1%) were classified as T0, 120 patients (75%) as T3, and 27 patients (16.9%) as T4. In terms of N stages, 112 patients (70%) were classified as N0, 39 patients (24.4%) as N1, and nine patients (5.6%) as N2. According to stage classification, 13 patients (8.1%) were classified as Stage 0, 81 patients (50.6%) as Stage II, and 66 patients (41.3%) as Stage III. Among the patients, 80 (50%) had squamous cell carcinoma, 70 (43.8%) had adenocarcinoma, and 10 (6.2%) had other types (large cell and adenosquamous cell carcinoma). The length of hospital stay was 5.4 ± 1.7 (range: 3–18) days in Group A and 5.1 ± 1.5 (range: 3–11) days in Group B, with no statistically significant difference (*p*: 0.142).

When comparing demographic and surgical outcomes by geriatric age group, the CCI was found to be “4 and above” in 97% of the geriatric age group, compared to 69.3% in those aged 70 and under (*p* < 0.001). Furthermore, among patients receiving neoadjuvant therapy, 42.1% were in Group A, while 23.9% were in Group B (*p* = 0.031). No statistically significant differences were found between the groups regarding other demographic characteristics (Table 1).

**TABLE 1:** Comparison of demographic and surgical characteristics by geriatric age group.

Variables		Group A (< 70 age)		Group B (≥ 70 age)		*p*‐Value
		*n*	%	*n*	%	
Gender	Male	109	95.6	43	93.5	0.575
	Female	5	4.4	3	6.5	
Smoking habit	Yes	15	13.2	9	19.6	0.304
	No	99	86.8	37	80.4	
CCI	< 4	35	30.9	1	2.2	**< 0.001**
	> 4	79	69.3	45	97.8	
Side	Right	69	60.5	26	56.5	0.641
	Left	45	39.5	20	43.5	
Superior sulcus tumor	No	54	47.4	26	56.5	0.295
	Yes	60	52.6	20	43.5	
Neoadjuvant treatment	No	66	57.9	35	76.1	**0.031**
	Yes	48	42.1	11	23.9	
Resections	Lobectomy	98	86	38	82.6	0.591
	Pneumonectomy	16	14	8	17.4	
T status	T0	12	10.5	1	2.2	0.181
	T3	82	71.9	38	82.6	
	T4	20	17.5	7	15.2	
N status	N0	81	71.1	31	67.4	0.182
	N1	29	25.4	10	21.7	
	N2	4	3.5	5	10.9	
Stage	0	12	10.5	1	2.2	0.196
	II	55	48.2	26	56.5	
	III	47	41.2	19	41.3	
Length of hospital stay		5.4 ± 1.7 (R: 3–18)		5.1 ± 1.5 (R: 3–11)		0.142
Complications		35	30.7	10	21.7	0.254
Mortality		7	6.1	2	4.3	1

A total of 45 patients (28.1%) were found to have complications; the complication profile is demonstrated in Table 2. When evaluating the factors affecting complications, no effect of the geriatric age group on complications was identified. There was no statistically significant difference in the development of complications with the increased number of rib resections in Group B. In Group A, however, cases undergoing resection of more than four ribs demonstrated a higher complication rate (*p* = 0.014). Prognostic factors affecting complications are evaluated in Table 3. In the study, mortality occurred in nine patients (5.6%) within the first 90 days. Among these mortalities, five were due to postoperative acute respiratory distress syndrome, three in patients who underwent pneumonectomy due to pneumonia following bronchopleural fistula, and one was due to air embolism. Of the patients who experienced mortality, seven (6.1%) were 70 years old or younger, and two patients (4.3%) were 70 years old or older. No statistically significant difference was found between the groups (*p* = 1).

**TABLE 2:** Postoperative complication profile.

	*N*	%
Prolonged air leak	14	8.8
Pneumonia	11	6.9
Cardiac arrhythmia	8	5
Empyema	5	3.1
Hemorrhage/hematoma	5	3.1
Bronchopleural fistula	4	2.5
Atelectasis	4	2.5
Wound infection	2	1.3
Metabolic disorders	2	1.3
Ptosis	1	0.6

**TABLE 3:** Variables affecting complications.

	Group A complications *N* (%)			Group B complications *N* (%)		
Variables	No	Yes	*p*	No	Yes	*p*
Male Female	75 (94.9) 4 (5.1)	34 (97.1) 1 (2.9)	0.596	33 (91.7) 3 (8.3)	10 (100) —	0.345
Smoking habit (Yes)	68 (86.1)	31 (88.6)	1	29 (80.6)	8 (80)	1
Right Left	50 (63.3) 29 (36.7)	19 (54.3) 16 (45.7)	0.364	19 (52.8) 17 (47.2)	7 (70) 3 (30)	0.331
Neoadjuvant treatment (Yes)	29 (36.7)	19 (54.3)	0.080	10 (27.8)	1 (10)	0.410
Superior sulcus tumor (yes)	37 (46.8)	23 (65.7)	0.063	15 (41.7)	5 (50)	0.638
Lobectomy Pneumonectomy	68 (86.1) 11 (13.9)	30 (85.7) 5 (14.3)	0.959	30 (83.3) 6 (16.7)	8 (80) 2 (20)	1
Mesh (yes)	12 (15.2)	8 (22.9)	0.321	5 (13.9)	3 (30)	0.344
Chest wall resection						
1–3 Ribs	59 (74.7)	18 (51.4)	0.014	27 (75)	8 (80)	1
> 4 Ribs	20 (25.3)	17 (48.6)		9 (25)	2 (20)	
T status						
T0	6 (7.6)	6 (17.1)		1 (2.8)	—	
T3	60 (75.9)	22 (62.9)	0.264	29 (80.6)	9 (90)	0.748
T4	13 (16.5)	7 (20)		6 (16.7)	1 (10)	
N status						
N0	55 (69.6)	26 (74.3)		25 (69.4)	6 (60)	
N1	20 (25.3)	9 (25.7)	0.505	8 (22.2)	2 (20)	0.561
N2	4 (5.1)	—		3 (8.3)	2 (20)	
Stage						
0	6 (7.6)	6 (17.1)		1 (2.8)	—	
II	40 (50.6)	15 (42.9)	0.312	21 (58.3)	5 (50)	0.780
III	33 (41.8)	14 (40)		14 (38.9)	5 (50)	

**FIGURE 1:**
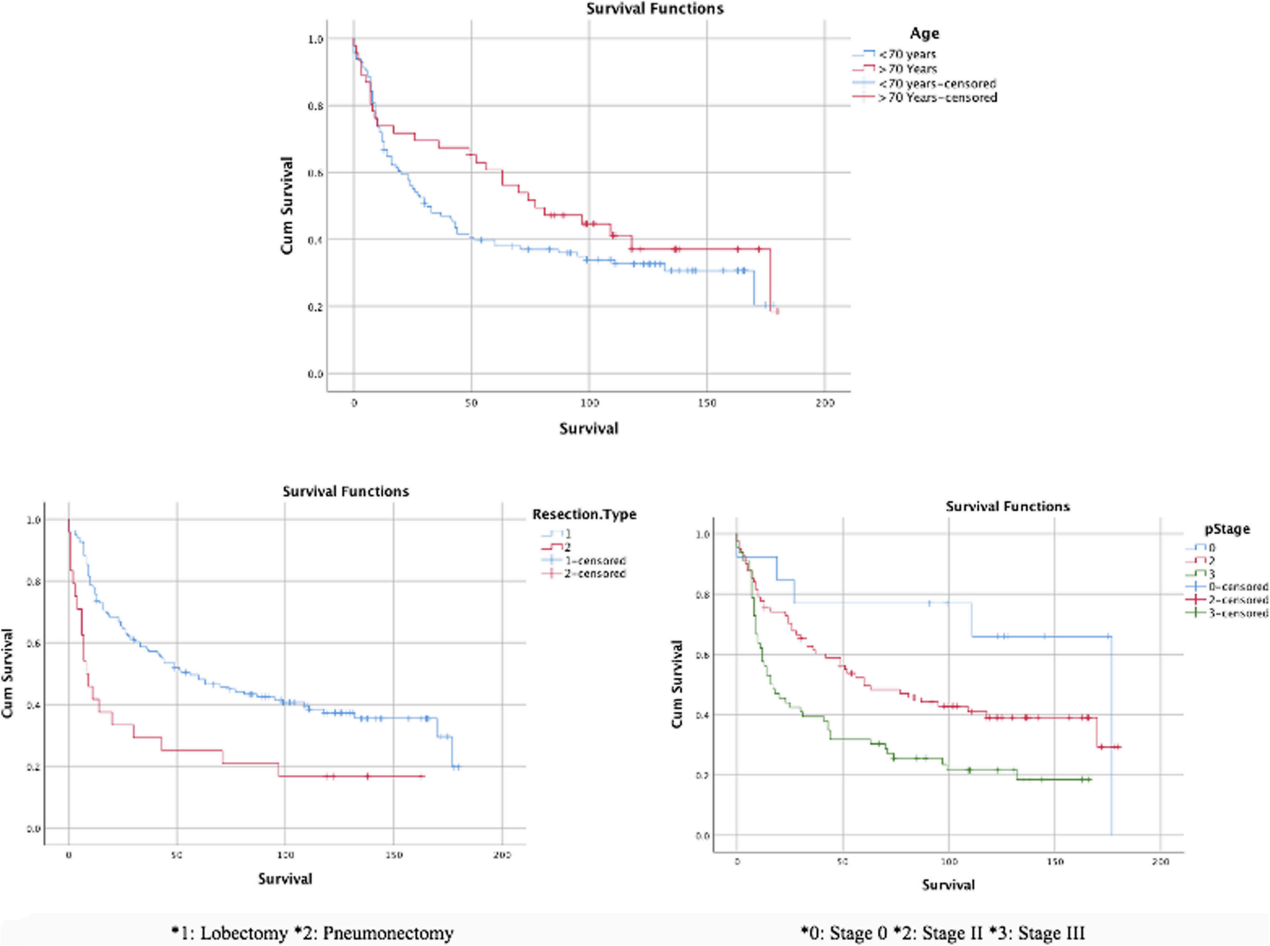
Survival analysis stratified by age group, surgical procedure (lobectomy vs. pneumonectomy), and pathological stage.

The average survival time of the patients was determined to be 79 months, with a 5‐year survival rate of 44.6%. When comparing age groups, the survival rate for Group A was 72%, while Group B had a survival rate of 93% (*p* = 0.189) (Figure 1). Regarding gender, the survival rate for males was 79%, while for females, it was 76% (*p* = 0.838). Patients with a CCI of ≤ 4 had a survival rate of 99%, compared to 72% for those with CCI > 4. No statistically significant difference was found between these groups (*p* = 0.055). According to histopathological results, the survival rate for patients diagnosed with adenocarcinoma was 88%, while the survival rate for those diagnosed with squamous cell carcinoma (SqCC) was 69% (*p* = 0.090). A complete pathological response (pCR) was achieved in 13 patients, representing 8.1%.

Prognostic factors affecting survival included the type of resection and p‐stage (Figure 1). When examining the type of resection, patients who underwent lobectomy had a survival rate of 85%, while those who had pneumonectomy had a survival rate of 41% (*p* < 0.001). In the geriatric age group, the 5‐year survival rate for patients receiving neoadjuvant therapy was 72.2%, while for those not receiving neoadjuvant therapy, it was 56.9% (*p* = 0.366). The average survival for patients with “pN0” status was 77 months; for “pN1” status, it was 14 months; and for “pN2” status, it was 8 months (*p* < 0.001). The evaluation of factors affecting survival is presented in Table 4. Multivariate analyses were performed separately in the two groups, and no increased risk was identified in relation to the number of rib resections. The details are provided in Table 5.

**TABLE 4:** Evaluation of factors affecting survival.

Variables		Overall survival (month)	%95 CI	5 Year survival (%)	*p*‐Value
Group A		72	59–86	38	0.189
Group B		93	72–114	60	
Gender	Male	79	67–91	44.3	0.838
	Female	76	30–122	50	
CCI	≤ 4	99	74–124	58.3	0.055
	> 4	72	59–85	40.5	
Side	Right	86	72–102	48.9	0.149
	Left	67	51–84	38.5	
Chest wall resection	1–3 Ribs	76	62–89	42.5	0.454
	> 4 Ribs	86	64–108	49.4	
Histopathology	SqCC	69	53–85	40.7	0.090
	Adenokarsinom	88	70–106	48.2	
Neoadjuvant treatment	No	78	63–92	45.4	0.753
	Yes	80	61–100	43	
Resections	Lobectomy	85	72	48	**< 0.001**
	Pneumonectomy	41	17	25	
Stage	yT0N0	103	90–174	76.9	**< 0.001**
	2	90	73–106	49.7	
	3	52	37–67	30.3	

Abbreviations: CCI, Charlson Comorbidity Index; CI, confidence interval; SqCC, squamous cell carcinoma.

**TABLE 5:** Multivariable analysis.

Variables	Group A			Group B		
	Hazard ratio	% 95 CI (lower–upper)	*p*‐Value	Hazard ratio	% 95 CI (lower–upper)	*p*‐Value
**Gender** Female/male	0.956	0.279–3.277	0.943	0.660	0.081–5.385	0.698
**Smoking habits** Yes/no	2.404	0.985–5.865	0.054	3.848	1.052–14.071	0.042
**Side** Right/left	0.989	0.585–1.672	0.966	0.601	0.211–1.716	0.342
**Neoadjuvant treatment** Yes/no	2.155	1.193–3.891	0.011	0.753	0.221–2.571	0.651
**Resection type** Lobectomy/pneumonectomy	0.638	0.290–1.400	0.262	0.524	0.147–1.869	0.319
**Superior sulcus tumor** Yes/no	1.853	0.959–3.579	0.066	0.381	0.125–1.159	0.089
**Chest wall resection** 1–3 Ribs/> 4 ribs	0.839	0.447–1.573	0.584	1.898	0.545–6.611	0.314
**T status**						
T0	2.038	0.416–9.978	0.082	2.146	0.073–63.065	0.895
T3	3.555	0.990–12.763	0.380	2.023	0.100–40.914	0.658
T4			0.052			0.646
**N status**						
N0	0.216	0.040–1.173	0.097	0.665	0.040–10.967	0.297
N1	0.741	0.191–2.882	0.076	0.272	0.048–1.548	0.776
N2			0.665			0.142
**Histopathology** Adenocarcinoma/SqCC	1.520	0.498–4.634	0.462	0.569	0.141–2.298	0.428
**Complications** Yes/no	1.690	0.976–2.925	0.061	0.903	0.293–2.786	0.859
**CCI group** **< 4/> 4**	0.752	0.388–1.455	0.397	0.000	0.000	0.982

## 4 Discussion

Until the early 2000s, surgical treatment for geriatric patients with lung cancer was controversial, and resection was generally avoided. In 2003, Peake et al. demonstrated in their multicenter study involving 1652 patients that elderly patients who could potentially undergo surgery were often undertreated, resulting in lower survival rates [7]. Over the past 20 years, numerous studies have shown that the survival outcomes of elderly patients undergoing surgery for lung cancer are comparable to those of younger patients, and that postoperative complications are at acceptable levels in appropriately selected patients [8]. Our study found no statistically significant differences in 5‐year survival and postoperative complication outcomes between patients aged 70 and older and those younger than 70.

The prospective study by Cerfolio et al. demonstrated that the 5‐year survival outcomes of patients aged 70 and older who underwent surgery for Stage 1 lung cancer were better compared to younger patients [9]. Furthermore, the study found that elderly patients receiving neoadjuvant therapy had a threefold increased risk of postoperative major morbidity. However, recent studies indicate that there is no association between neoadjuvant treatment and an increase in major complications in elderly patients. Our study also observed that neoadjuvant therapy did not increase postoperative complications [10]. The lower incidence of complications in surgeries following neoadjuvant treatment in current studies could be attributed to increased surgical experience.

One of the key advantages of neoadjuvant therapy is the identification of complete pathological response (pCR). After achieving a complete pathological response, the 5‐year overall survival rates are reported to be between 70% and 90% [11, 12]. The study by Jones et al. found that patients who achieved a complete response had better survival outcomes than those who did not respond or showed progression (HR = 3.5, *p* = 0.002) [13]. In this study, the 2‐year survival rate for patients with pCR was found to be 74%. Kawaguchi et al., in their prospective neoadjuvant treatment study (CJSG0801), reported a 3‐year survival rate of 91.7% (*p* = 0.039) in patients who achieved a complete response after induction therapy [12]. We previously reported a general survival rate of 73.8 ± 7.2 (95% CI: 59.6–88.0) following complete pathological response in patients with NSCLC [14]. In our current study, the pCR rate was found to be %8.1. In cases of NSCLC with chest wall involvement, lymph node metastasis is considered one of the most important prognostic factors. The role of surgery after induction therapy in patients with N2 superior sulcus tumors is still debated. Jones et al. identified pN2 status as the most significant prognostic factor affecting survival in patients with NSCLC and chest wall invasion. In the study by Chapelier et al., the survival rate for pN0 patients was 22%, while it was 9% for pN1 patients [15]. In the study by Facciolo et al., the 5‐year survival rate for pN0 patients was 67%, whereas it was only 18% for N2 patients with chest wall invasion [16]. Similarly, Doddoli et al. reported a 5‐year survival rate of 8% for pN2 patients and 40% for pN0 patients [17]. Matsuoka et al. found that the 5‐year survival rate for pN0 patients was 44%, while only 6.2% for pN2 patients [18]. In our study, the pN2 was 77 months, and the results for pN0 patients were found to be 8, consistent with the literature. We emphasize the importance of thoroughly evaluating nodal disease status in the preoperative period.

Lung cancer with chest wall invasion is a type of locally advanced stage that can be surgically treated. While resection of the chest wall in conjunction with lung resection has shown promising survival outcomes, it has been well established for many years that there is an acceptable increase in postoperative morbidity [19]. However, the additional postoperative morbidities that may arise from chest wall resection in elderly patients have led many surgeons to avoid such procedures. Consequently, limited studies in the literature examine the outcomes of chest wall resection in elderly patients. In a study conducted by Weyant et al. in 2006, it was reported that elderly patients experienced a higher incidence of respiratory complications, with pneumonectomy being identified as a risk factor [20]. More recent studies, such as that by Hayashi et al., demonstrated that when a thorough preoperative cardiopulmonary assessment is conducted, elderly patients experience a similar rate of complications as younger patients undergoing chest wall resection. In this study, pneumonectomy, resection of three or more ribs, and the amount of intraoperative bleeding were identified as risk factors for postoperative complications in patients undergoing chest wall resection [21].

Furthermore, Sezen et al. showed that postoperative complication rates and survival values in elderly patients undergoing chest wall resection were similar to those in younger patients [22]. They also noted that a high American Society of Anesthesiologists score was a risk factor for postoperative complications, emphasizing the importance of appropriate patient selection for surgery. Our study's results align with the literature, indicating that elderly patients do not differ significantly from younger patients regarding survival and the risk of developing postoperative complications [22]. When examining risk factors for postoperative complications, we observed that resection of four or more ribs was the only significant risk factor. Contrary to the general literature, pneumonectomy did not significantly increase complications in our study. This finding can be attributed to good preoperative patient selection. However, pneumonectomy and advanced stage were found to be associated with poorer survival outcomes, as expected. A CCI value of four or more was not identified as a risk factor for the development of complications, although it was observed to be partially disadvantageous for survival; however, this was not statistically significant.

Several methods for accurate preoperative assessment in elderly patients have been described in the literature. In addition to high ASA and CCI scores and a predicted postoperative FEV1 < 55%, factors such as low body mass index, being an active smoker, and having emphysematous or interstitial lung patterns have also been evaluated as risk factors for postoperative complications in elderly patients [23].

### 4.1 Limitations

The study's limitations include its retrospective nature, the small number of female patients, and the constraints of morbidity scoring systems that could contribute to complications in patients' preoperative evaluations.

## 5 Conclusion

Based on the data from our study, no significant prognostic factors were identified for chest wall resections in geriatric age groups with non‐small cell lung cancer; however, the width of resection and stage were found to be important factors for survival. These findings indicate that challenging surgeries, such as chest wall resection, can be safely performed in appropriately selected elderly patients, yielding positive outcomes similar to those in younger patients.

## Author Contributions

Conceptualization: Celal Bugra Sezen, Ozkan Saydam, Mustafa Vedat Dogru. Data curation: Umut Kilimci, Oğuzhan Bayraktar. Formal analysis: Celal Buğra Sezen. Funding acquisition: Cemal Aker. Investigation: Özkan Saydam. Methodology: Özkan Saydam. Project administration: Cemal Aker, Mustafa Vedat Doğru. Resources: Melike Ülker. Software: Celal Buğra Sezen. Supervision: Muzaffer Metin. Validation: Melike Ülker. Writing – original draft: Özkan Saydam, Celal Buğra Sezen. Writing – review and editing: Özkan Saydam, Melike Ülker.

## Ethics Statement

This study was conducted in accordance with the Declaration of Helsinki. Ethical approval was obtained from the Institutional Review Board (Approval Number: 2021‐103).

## Consent

A written informed consent was obtained from each patient.

## Conflicts of Interest

The authors declare no conflicts of interest.

## Data Availability

The data that support the findings of this study are available from the corresponding author upon reasonable request.
